# Crystal structure determination as part of an undergraduate laboratory experiment: 1′,3′,3′-tri­methyl­spiro­[chromene-2,2′-indoline] and 1′,3′,3′-trimethyl-4-[(*E*)-(1,3,3-tri­methyl­indolin-2-yl­idene)meth­yl]spiro­[chroman-2,2′-indoline]

**DOI:** 10.1107/S2056989016016042

**Published:** 2016-10-28

**Authors:** Joseph O. S. Beckett, Marilyn M. Olmstead, James C. Fettinger, David A. Gray, Shuhei Manabe, Mark Mascal

**Affiliations:** a Department of Chemistry, University of California, Davis, One Shields Avenue, Davis, CA 95616, USA

**Keywords:** crystal structure, spiro­pyran, undergraduate teaching laboratory

## Abstract

The crystal structures of the title compounds, C_19_H_19_NO and C_31_H_34_N_2_O, were determined as part of an experiment in an undergraduate teaching laboratory that demonstrates the relationship between mol­ecular structure and function. 1′,3′,3′-Tri­methyl­spiro­[chromene-2,2′-indoline] is both a photoswitch and thermochromic mol­ecule. Students synthesized it and a bis-indoline adduct and compared the crystallographically determined structures to computed gas-phase models.

## Abstract 2

The crystal structures of the title compounds were determined as part of an experiment in an undergraduate teaching laboratory that demonstrates the relationship between mol­ecular structure and function. 1′,3′,3′-Tri­methyl­spiro­[chromene-2,2′-indoline] is both a photoswitch and thermochromic mol­ecule. Students synthesized it and a bis-indoline adduct and compared the crystallographically determined structures to computed gas-phase models.

## Chemical context   

In an ever evolving pursuit to improve the educational experience in undergraduate organic chemistry laboratory courses, we introduced an experiment in which students prepare a ‘functional mol­ecule,’ in this case spiro­pyran **1**. Compounds such as **1** are broadly characterized as ‘responsive,’ due to their ability to be actuated by a range of stimuli, including light, heat, metal ions, pH, mechanical force, and changes in solvent polarity (Klajn, 2014 ▸). An advantage of the spiro­pyran system over other photochromic/thermochromic materials is the strongly differentiated electronic forms between which equilibrium is shifted. The closed-ring isomer of **1** comprises an indoline and a chromene ring bound together at a spiro junction, while the open-ring form is a zwitterionic merocyanine **1** ***a*** (Scheme 1).
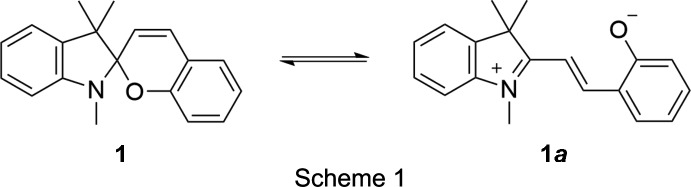


Although a variety of substituted spiro­pyran derivatives are known in the literature, for simplicity, we elected to focus on the unsubstituted parent compound, which is colorless in its closed form and red in its open form. The mol­ecule was synthesized in a single step by condensation of 1,3,3-trimethyl-2-methyl­eneindoline with salicyl­aldehyde (Koelsch & Workman, 1952 ▸). The methyl­eneindoline nucleophile can also react a second time with **1** to give the bis adduct **2** as a side product (Scheme 2).

Since this experiment was oriented around the functional attributes of **1**, it presented an ideal opportunity to introduce structural characterization methods into the laboratory course, since the function of **1** is directly linked to its structure. Students first model the two forms of **1** using both mol­ecular mechanics and semi-empirical quantum mechanical methods. These calculations indicate that the spiro­pyran form of **1** is more stable than the open form **1** ***a***. They then grow crystals of **1** by slow evaporation from acetone, resulting in most cases in large (up to 10 mm × 10 mm), thin pink plates. Although the students do not themselves determine the X-ray crystal structure, crystallographic characterization of **1** has allowed students to compare gas-phase models with condensed-state empirical data.
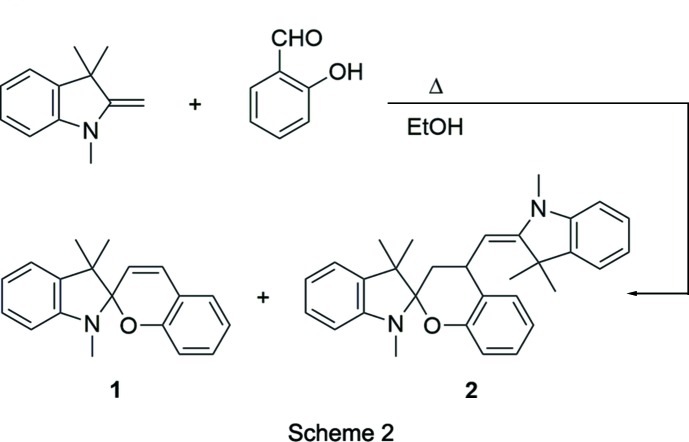
2

## Structural commentary   

**Figure 1:**
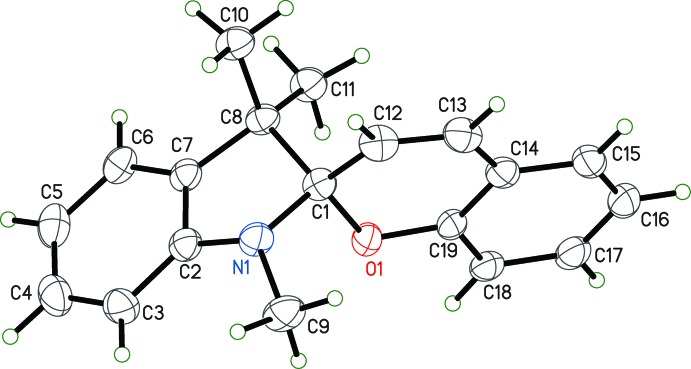
The mol­ecular structure of **1**. Displacement parameters are shown at the 50% probability level.

**Figure 2:**
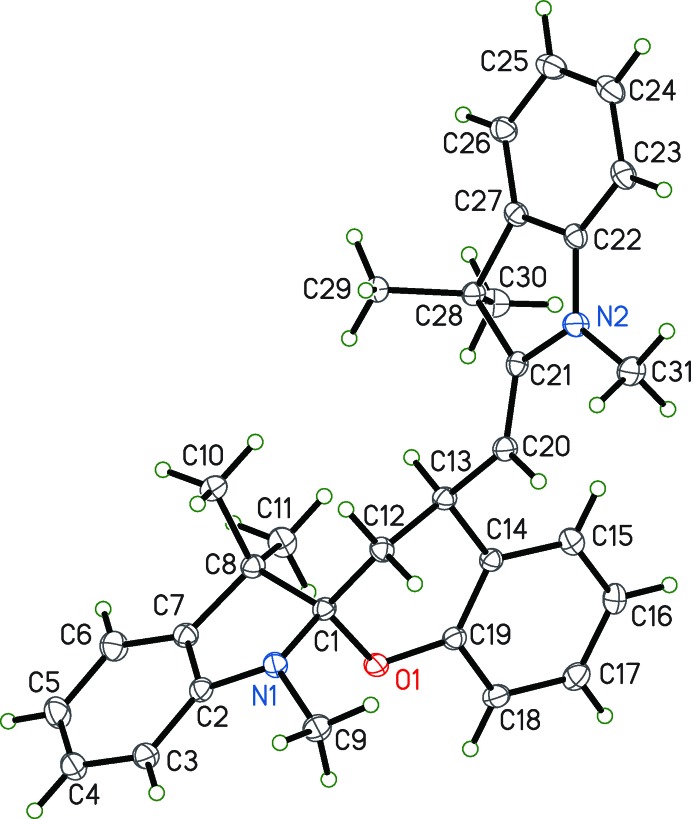
The mol­ecular structure of **2**. Displacement parameters are shown at the 50% probability level.

Crystals of the parent spiro­pyran, 1′,3′,3′-tri­methyl­spiro[chro­mene-2,2′-indoline] **1**, are colorless at low temperature (90 K). Fig. 1 ▸ depicts the low-temperature crystal structure. There is one mol­ecule in the asymmetric unit. The central *sp* ^3^ carbon atom, C1, has a tetra­hedral geometry. The dihedral angle between O1/C1/C12 and N1/C1/C8 is 89.33 (12)°. The C12—C13 bond is a double bond with a length of 1.330 (3) Å. The substituted spiro­pyran, 1′,3′,3′-trimethyl-4-[(*E*)-(1,3,3-trimethyl­indolin-2-yl­idene)meth­yl]spiro­[chroman-2,2′-indoline] **2**, is also colorless at low temperature. It differs from **1** by virtue of substitution at C13 with a methyl­eneindoline group (Fig. 2 ▸). Consequently, C12 and C13 are now singly bonded, with a distance of 1.5367 (14) Å. The central carbon atom remains tetra­hedral with the value of the dihedral angle at 89.69 (5), comparable to **1**. The atoms C1 and C13 have the same chirality, either *RR* or *SS*.

Differences between mol­ecular mechanics force field MM2 calculations and the semi-empirical quantum mechanical methods PM6 and PDDG *versus* experimental X-ray values for selected bond lengths and angles can be seen in Table 1 ▸. A clear trend in the data is reflected in the fact that thermal motion in low-temperature X-ray diffraction experiments tends to lead to an apparent bond shortening. Considering only those distances not involving phenyl carbon atoms, the data indicate that MM2 shows the poorest mean agreement with X-ray in bond lengths (±0.043 Å), while PDDG (±0.021 Å) and PM6 (±0.017 Å) perform better. The most serious modeling failure was in the MM2 N1—C2 bond which, at 1.270 Å, was inter­preted by mol­ecular mechanics to be a double bond, but which was clearly a single bond in the X-ray structure at 1.405 (2) Å. As a consequence, the sum of the angles at N1 was 360° in the MM2 calculation, whereas the experimental value was 348.36°. PM6 and PDDG again performed better here, with sums of 345.4 and 344.5°, respectively. The dihedral angle between the O1/C1/C2 plane and the N1/C1/C8 plane was 89.3° for X-ray, compared to 92.7° for MM2, 91.3° for PM6 and 91.4° for PDDG. Bond angle deviations ranged from 0 to 5° and averaged *ca* 2° for all three methods. Inter­estingly, if the two angles in poor agreement around C1 are discarded, MM2 actually performs somewhat better than the semi-empirical models for angle data. If all data in Table 1 ▸ are taken into account, PM6 is seen to outperform both PDDG and MM2.

## Supra­molecular features   

The KPI of **1** is 68.7% and that of **2** is 69.6% (van der Sluis & Spek, 1990 ▸). Neither structure has significant directional inter­molecular inter­actions.

## Database survey   

There are 67 structures in the CSD (Groom *et al.*, 2016 ▸) with the basic skeleton of compound **1**. All of these are substituted in one way or another. There are no unusual differences among these structures. Since the C1—O1 bond is broken in the transformation to the merocyanine form, it is of inter­est to examine this bond length. Of the 82 hits with similar geometry, the mean C—O distance in the CSD is 1.479 (15)°. For **1**, this distance is 1.4708 (19) Å. For **2**, the same distance is 1.4648 (12) Å. There are five structures in the CSD that involve further methyl­eneindoline substitution, similar to **2**. In all cases, the structures are racemic and the chirality is either *RR* or *SS*. Two of the deposits (NESZOC and NESZOC01; Ashraf *et al.*, 2012 ▸) describe the results from two different crystals, two different radiations (Cu *K*α and Mo *K*α), and two different temperatures (153 and 113 K), respectively. Structurally, there is no significant difference between them, but the higher temperature crystal is described as a red prism while the lower temperature crystal is a pink plate. This feature was not discussed, but it raises the possibility of a merocyanine impurity arising due to the thermochromic effect.

## Synthesis and crystallization   

A solution of 1,3,3-trimethyl-2-methyl­eneindoline (3.37 g, 19.5 mmol) and salicyl­aldehyde (2.53 g, 20.7 mmol) in absolute ethanol (15 mL) was heated at reflux with stirring for 1 h. A white precipitate was filtered from the hot solution and washed with cold absolute ethanol. The solid was recrystallized from acetone to give 1′,3′,3′-trimethyl-4-[(*E*)-(1,3,3-tri­methyl­indolin-2-yl­idene)meth­yl]spiro­[chroman-2,2′-indoline] **2** (0.49 g, 11%), m.p. 474–477 K. The filtrate/wash was then evaporated and the residue was recrystallized from 90% ethanol to give 1′,3′,3′-tri­methyl­spiro­[chromene-2,2′-indoline] **1** (2.58 g, 48%), m.p. 366-368 K. Crystals of **1** and **2** suitable for X-ray diffraction were obtained by slow evaporation from acetone solutions.

## Refinement   

Crystal data, data collection and structure refinement details are summarized in Table 2 ▸. The hydrogen atoms bonded to carbon were located by geometry and refined using a riding model. Distances were fixed at 0.95 Å for C—H bonds in phenyl rings and 0.98 Å in methyl groups. In structure **2**, primary C—H bonds were assigned C—H distances of 1.00 Å while secondary C—H distances were given values of 0.99 Å. The *U*_iso_(H) parameters were set equal to 1.5*U*_eq_ for the methyl groups and to 1.2*U*_eq_ of the parent carbon for all others.

## Supplementary Material

Crystal structure: contains datablock(s) 1, 2. DOI: 10.1107/S2056989016016042/hb7609sup1.cif

Structure factors: contains datablock(s) 1. DOI: 10.1107/S2056989016016042/hb76091sup4.hkl

Structure factors: contains datablock(s) 2. DOI: 10.1107/S2056989016016042/hb76092sup3.hkl

Supporting information file. DOI: 10.1107/S2056989016016042/hb76091sup4.cml

Supporting information file. DOI: 10.1107/S2056989016016042/hb76092sup5.cml

CCDC references: 1509185, 1509184

Additional supporting information:  crystallographic information; 3D view; checkCIF report

## Figures and Tables

**Table 1:** Comparison of modeled (MM2, PDDG, PM6) bond lengths, angles, and dihedral angles (Å, °) with X-ray crystallographic data

	X-ray	MM2	Δ	PDDG	Δ	PM6	Δ
C1—O1	1.471	1.415	0.056	1.423	0.048	1.484	−0.013
C1—N1	1.447	1.488	−0.041	1.515	−0.068	1.493	−0.046
C1—C8	1.580	1.588	−0.008	1.589	−0.009	1.599	−0.019
C1—C12	1.496	1.508	−0.012	1.504	−0.008	1.497	−0.001
N1—C2	1.405	1.270	0.135	1.428	−0.023	1.430	−0.025
N1—C9	1.457	1.475	−0.018	1.468	−0.011	1.481	−0.024
C12—C13	1.330	1.338	−0.008	1.340	−0.010	1.340	−0.010
C13—C14	1.453	1.343	0.110	1.448	0.005	1.455	−0.002
O1—C19	1.370	1.368	0.002	1.366	0.004	1.362	0.008
							
**|mean|**			0.043		0.021		0.017
							
Dihedral angle O1/C1/C12 and N1/C1/C8	89.33	92.7	−3.370	91.4	−2.070	91.3	−1.970
Sum of angles at N1	348.36	360.0	−11.640	345.4	2.960	344.5	3.860
							
C1—O1—C19	121.03	119.1	1.93	118.4	2.63	121.3	−0.27
O1—C1—C12	111.35	111.3	0.05	115.4	−4.05	113.7	−2.35
O1—C1—C8	108.57	109.1	−0.53	110.2	−1.63	104.8	3.77
N1—C1—C8	102.85	104.3	−1.45	104.9	−2.05	105.3	−2.45
N1—C1—O1	105.75	110.3	−4.55	103.9	1.85	104.5	1.25
N1—C1—C12	112.92	107.6	5.32	109.1	3.82	111.0	1.92
C8—C1—C12	114.70	114.0	0.70	112.3	2.40	116.6	−1.90
							
**|mean|**			2.08		2.63		1.99

**Table 2:** Experimental details

	**1**	**2**
Crystal data		
Chemical formula	C_19_H_19_NO	C_31_H_34_N_2_O
*M*_r_	277.35	450.60
Crystal system, space group	Monoclinic, *P*2_1_/*c*	Monoclinic, *P*2_1_/*c*
Temperature (K)	90	90
*a*, *b*, *c* (Å)	11.530 (7), 10.938 (6), 13.013 (7)	14.1774 (11), 11.6019 (9), 16.2847 (17)
β (°)	115.614 (7)	115.6129 (12)
*V* (Å^3^)	1479.9 (15)	2415.4 (4)
*Z*	4	4
Radiation type	Mo *K*α	Mo *K*α
μ (mm^−1^)	0.08	0.07
Crystal size (mm)	0.52 × 0.36 × 0.35	0.48 × 0.26 × 0.08

Data collection		
Diffractometer	Bruker SMART 1000	Bruker DUO
Absorption correction	Multi-scan (*SADABS*; Bruker, 2014 ▸)	Multi-scan (*SADABS*; Bruker, 2014 ▸)
*T*_min_, *T*_max_	0.811, 0.983	0.713, 0.746
No. of measured, independent and observed [*I* > 2σ(*I*)] reflections	12543, 3358, 2672	39237, 7680, 6549
*R*_int_	0.029	0.026
(sin θ/λ)_max_ (Å^−1^)	0.650	0.725

Refinement		
*R*[*F* ^2^ > 2σ(*F* ^2^)], *wR*(*F* ^2^), *S*	0.050, 0.139, 1.05	0.046, 0.124, 1.03
No. of reflections	3358	7680
No. of parameters	193	313
H-atom treatment	H-atom parameters constrained	H-atom parameters constrained
Δρ_max_, Δρ_min_ (e Å^−3^)	0.23, −0.23	0.61, −0.22

Computer programs: *SMART* (Bruker, 2002 ▸), *SAINT* (Bruker, 2013 ▸, 2014 ▸), *APEX2* (Bruker, 2014 ▸), *SHELXTL* (Sheldrick, 2008 ▸), *SHELXT* (Sheldrick, 2015*a* ▸) and *SHELXL2014* (Sheldrick, 2015*b* ▸).

## supplementary crystallographic information

### (1) 1',3',3'-Trimethylspiro[chromene-2,2'-indoline] Crystal data

**Table:**

C_19_H_19_NO	*F*(000) = 592
*M**_r_* = 277.35	*D*_x_ = 1.245 Mg m^−^^3^
Monoclinic, *P*2_1_/*c*	Mo *K*α radiation, λ = 0.71073 Å
*a* = 11.530 (7) Å	Cell parameters from 9931 reflections
*b* = 10.938 (6) Å	θ = 2.6–27.4°
*c* = 13.013 (7) Å	µ = 0.08 mm^−^^1^
β = 115.614 (7)°	*T* = 90 K
*V* = 1479.9 (15) Å^3^	Block, colorless
*Z* = 4	0.52 × 0.36 × 0.35 mm

### (1) 1',3',3'-Trimethylspiro[chromene-2,2'-indoline] Data collection

**Table:**

Bruker SMART 1000 diffractometer	3358 independent reflections
Radiation source: fine-focus sealed tube	2672 reflections with *I* > 2σ(*I*)
Detector resolution: 8.3 pixels mm^-1^	*R*_int_ = 0.029
ω scans	θ_max_ = 27.5°, θ_min_ = 2.0°
Absorption correction: multi-scan (SADABS; Bruker, 2014)	*h* = −14→14
*T*_min_ = 0.811, *T*_max_ = 0.983	*k* = −14→14
12543 measured reflections	*l* = −16→16

### (1) 1',3',3'-Trimethylspiro[chromene-2,2'-indoline] Refinement

**Table:**

Refinement on *F*^2^	Primary atom site location: dual
Least-squares matrix: full	Secondary atom site location: difference Fourier map
*R*[*F*^2^ > 2σ(*F*^2^)] = 0.050	Hydrogen site location: inferred from neighbouring sites
*wR*(*F*^2^) = 0.139	H-atom parameters constrained
*S* = 1.05	*w* = 1/[σ^2^(*F*_o_^2^) + (0.0554*P*)^2^ + 1.046*P*] where *P* = (*F*_o_^2^ + 2*F*_c_^2^)/3
3358 reflections	(Δ/σ)_max_ < 0.001
193 parameters	Δρ_max_ = 0.23 e Å^−^^3^
0 restraints	Δρ_min_ = −0.23 e Å^−^^3^

### (1) 1',3',3'-Trimethylspiro[chromene-2,2'-indoline] Special details

**Table:**

Geometry. All esds (except the esd in the dihedral angle between two l.s. planes) are estimated using the full covariance matrix. The cell esds are taken into account individually in the estimation of esds in distances, angles and torsion angles; correlations between esds in cell parameters are only used when they are defined by crystal symmetry. An approximate (isotropic) treatment of cell esds is used for estimating esds involving l.s. planes.

### (1) 1',3',3'-Trimethylspiro[chromene-2,2'-indoline] Fractional atomic coordinates and isotropic or equivalent isotropic displacement parameters (Å^2^)

**Table:**

	*x*	*y*	*z*	*U*_iso_*/*U*_eq_	
O1	0.80339 (11)	0.34663 (10)	0.35758 (9)	0.0279 (3)	
N1	0.80594 (13)	0.55031 (13)	0.30705 (12)	0.0296 (3)	
C1	0.73425 (15)	0.43762 (14)	0.26886 (13)	0.0271 (3)	
C2	0.76732 (15)	0.60633 (14)	0.38435 (14)	0.0284 (3)	
C3	0.83102 (17)	0.69436 (16)	0.46624 (15)	0.0362 (4)	
H3	0.9137	0.7230	0.4784	0.043*	
C4	0.76958 (19)	0.73941 (16)	0.53015 (16)	0.0392 (4)	
H4	0.8110	0.8002	0.5863	0.047*	
C5	0.64926 (19)	0.69733 (16)	0.51348 (15)	0.0373 (4)	
H5	0.6089	0.7298	0.5576	0.045*	
C6	0.58729 (16)	0.60716 (15)	0.43184 (14)	0.0310 (4)	
H6	0.5050	0.5777	0.4203	0.037*	
C7	0.64723 (15)	0.56145 (14)	0.36826 (13)	0.0267 (3)	
C8	0.60256 (14)	0.46823 (14)	0.27321 (13)	0.0261 (3)	
C9	0.93940 (16)	0.55554 (18)	0.32437 (17)	0.0386 (4)	
H9A	0.9487	0.5105	0.2633	0.058*	
H9B	0.9948	0.5187	0.3981	0.058*	
H9C	0.9645	0.6410	0.3234	0.058*	
C10	0.50907 (16)	0.52895 (16)	0.16130 (14)	0.0329 (4)	
H10A	0.4821	0.4690	0.0993	0.049*	
H10B	0.5521	0.5976	0.1437	0.049*	
H10C	0.4335	0.5590	0.1696	0.049*	
C11	0.53771 (16)	0.35560 (15)	0.29460 (15)	0.0312 (4)	
H11A	0.5220	0.2952	0.2344	0.047*	
H11B	0.4557	0.3794	0.2945	0.047*	
H11C	0.5938	0.3199	0.3687	0.047*	
C12	0.72190 (17)	0.39690 (17)	0.15482 (14)	0.0336 (4)	
H12	0.7053	0.4565	0.0970	0.040*	
C13	0.73330 (16)	0.28041 (17)	0.13139 (14)	0.0339 (4)	
H13	0.7175	0.2579	0.0560	0.041*	
C14	0.76943 (15)	0.18690 (15)	0.21904 (14)	0.0290 (3)	
C15	0.77489 (16)	0.06223 (16)	0.19799 (16)	0.0343 (4)	
H15	0.7521	0.0349	0.1224	0.041*	
C16	0.81308 (16)	−0.02187 (16)	0.28582 (17)	0.0372 (4)	
H16	0.8147	−0.1066	0.2704	0.045*	
C17	0.84899 (15)	0.01812 (16)	0.39650 (16)	0.0344 (4)	
H17	0.8755	−0.0395	0.4570	0.041*	
C18	0.84650 (14)	0.14200 (15)	0.41974 (14)	0.0290 (3)	
H18	0.8735	0.1692	0.4960	0.035*	
C19	0.80426 (14)	0.22559 (14)	0.33084 (13)	0.0261 (3)	

### (1) 1',3',3'-Trimethylspiro[chromene-2,2'-indoline] Atomic displacement parameters (Å^2^)

**Table:**

	*U*^11^	*U*^22^	*U*^33^	*U*^12^	*U*^13^	*U*^23^
O1	0.0296 (6)	0.0243 (5)	0.0275 (6)	0.0041 (4)	0.0103 (5)	0.0002 (4)
N1	0.0238 (7)	0.0291 (7)	0.0385 (8)	−0.0025 (5)	0.0159 (6)	0.0002 (6)
C1	0.0259 (7)	0.0271 (8)	0.0285 (8)	0.0003 (6)	0.0119 (6)	0.0029 (6)
C2	0.0269 (8)	0.0243 (7)	0.0331 (8)	0.0008 (6)	0.0122 (7)	0.0031 (6)
C3	0.0346 (9)	0.0276 (8)	0.0402 (9)	−0.0035 (7)	0.0104 (7)	−0.0001 (7)
C4	0.0511 (11)	0.0254 (8)	0.0361 (9)	0.0006 (7)	0.0142 (8)	−0.0005 (7)
C5	0.0509 (11)	0.0284 (8)	0.0362 (9)	0.0089 (8)	0.0223 (8)	0.0036 (7)
C6	0.0318 (8)	0.0278 (8)	0.0360 (8)	0.0057 (6)	0.0172 (7)	0.0065 (7)
C7	0.0266 (7)	0.0234 (7)	0.0292 (8)	0.0028 (6)	0.0112 (6)	0.0043 (6)
C8	0.0230 (7)	0.0271 (8)	0.0279 (8)	0.0000 (6)	0.0106 (6)	0.0037 (6)
C9	0.0254 (8)	0.0435 (10)	0.0490 (10)	−0.0023 (7)	0.0180 (8)	0.0036 (8)
C10	0.0282 (8)	0.0340 (9)	0.0333 (9)	0.0012 (7)	0.0102 (7)	0.0059 (7)
C11	0.0280 (8)	0.0295 (8)	0.0363 (9)	−0.0024 (6)	0.0143 (7)	0.0036 (7)
C12	0.0338 (9)	0.0399 (9)	0.0291 (8)	−0.0008 (7)	0.0154 (7)	0.0027 (7)
C13	0.0315 (8)	0.0438 (10)	0.0287 (8)	−0.0027 (7)	0.0151 (7)	−0.0041 (7)
C14	0.0218 (7)	0.0350 (9)	0.0317 (8)	−0.0017 (6)	0.0130 (6)	−0.0055 (7)
C15	0.0244 (8)	0.0383 (9)	0.0405 (9)	−0.0031 (7)	0.0144 (7)	−0.0123 (7)
C16	0.0259 (8)	0.0298 (9)	0.0528 (11)	−0.0002 (7)	0.0143 (8)	−0.0079 (8)
C17	0.0226 (8)	0.0298 (8)	0.0474 (10)	0.0014 (6)	0.0118 (7)	0.0021 (7)
C18	0.0204 (7)	0.0317 (8)	0.0330 (8)	0.0017 (6)	0.0096 (6)	0.0007 (7)
C19	0.0196 (7)	0.0268 (8)	0.0330 (8)	0.0007 (6)	0.0125 (6)	−0.0037 (6)

### (1) 1',3',3'-Trimethylspiro[chromene-2,2'-indoline] Geometric parameters (Å, º)

**Table:**

O1—C19	1.370 (2)	C9—H9C	0.9800
O1—C1	1.4708 (19)	C10—H10A	0.9800
N1—C2	1.405 (2)	C10—H10B	0.9800
N1—C1	1.447 (2)	C10—H10C	0.9800
N1—C9	1.457 (2)	C11—H11A	0.9800
C1—C12	1.496 (2)	C11—H11B	0.9800
C1—C8	1.580 (2)	C11—H11C	0.9800
C2—C3	1.388 (2)	C12—C13	1.330 (3)
C2—C7	1.397 (2)	C12—H12	0.9500
C3—C4	1.395 (3)	C13—C14	1.452 (2)
C3—H3	0.9500	C13—H13	0.9500
C4—C5	1.387 (3)	C14—C19	1.397 (2)
C4—H4	0.9500	C14—C15	1.398 (2)
C5—C6	1.398 (3)	C15—C16	1.382 (3)
C5—H5	0.9500	C15—H15	0.9500
C6—C7	1.381 (2)	C16—C17	1.386 (3)
C6—H6	0.9500	C16—H16	0.9500
C7—C8	1.511 (2)	C17—C18	1.391 (2)
C8—C11	1.528 (2)	C17—H17	0.9500
C8—C10	1.538 (2)	C18—C19	1.387 (2)
C9—H9A	0.9800	C18—H18	0.9500
C9—H9B	0.9800		
			
C19—O1—C1	121.03 (12)	H9A—C9—H9C	109.5
C2—N1—C1	107.85 (13)	H9B—C9—H9C	109.5
C2—N1—C9	120.85 (14)	C8—C10—H10A	109.5
C1—N1—C9	119.66 (14)	C8—C10—H10B	109.5
N1—C1—O1	105.75 (12)	H10A—C10—H10B	109.5
N1—C1—C12	112.92 (14)	C8—C10—H10C	109.5
O1—C1—C12	111.35 (13)	H10A—C10—H10C	109.5
N1—C1—C8	102.85 (13)	H10B—C10—H10C	109.5
O1—C1—C8	108.57 (12)	C8—C11—H11A	109.5
C12—C1—C8	114.70 (13)	C8—C11—H11B	109.5
C3—C2—C7	121.35 (16)	H11A—C11—H11B	109.5
C3—C2—N1	128.78 (16)	C8—C11—H11C	109.5
C7—C2—N1	109.87 (14)	H11A—C11—H11C	109.5
C2—C3—C4	117.80 (17)	H11B—C11—H11C	109.5
C2—C3—H3	121.1	C13—C12—C1	122.43 (16)
C4—C3—H3	121.1	C13—C12—H12	118.8
C5—C4—C3	121.40 (17)	C1—C12—H12	118.8
C5—C4—H4	119.3	C12—C13—C14	121.19 (16)
C3—C4—H4	119.3	C12—C13—H13	119.4
C4—C5—C6	120.06 (17)	C14—C13—H13	119.4
C4—C5—H5	120.0	C19—C14—C15	118.85 (16)
C6—C5—H5	120.0	C19—C14—C13	117.40 (15)
C7—C6—C5	119.14 (16)	C15—C14—C13	123.71 (16)
C7—C6—H6	120.4	C16—C15—C14	120.83 (17)
C5—C6—H6	120.4	C16—C15—H15	119.6
C6—C7—C2	120.22 (16)	C14—C15—H15	119.6
C6—C7—C8	130.76 (15)	C15—C16—C17	119.62 (17)
C2—C7—C8	108.96 (14)	C15—C16—H16	120.2
C7—C8—C11	114.54 (14)	C17—C16—H16	120.2
C7—C8—C10	109.56 (13)	C16—C17—C18	120.55 (17)
C11—C8—C10	108.83 (13)	C16—C17—H17	119.7
C7—C8—C1	100.34 (12)	C18—C17—H17	119.7
C11—C8—C1	112.92 (13)	C19—C18—C17	119.56 (16)
C10—C8—C1	110.41 (13)	C19—C18—H18	120.2
N1—C9—H9A	109.5	C17—C18—H18	120.2
N1—C9—H9B	109.5	O1—C19—C18	117.61 (14)
H9A—C9—H9B	109.5	O1—C19—C14	121.79 (15)
N1—C9—H9C	109.5	C18—C19—C14	120.54 (15)
			
C2—N1—C1—O1	−82.59 (15)	C2—C7—C8—C1	18.11 (16)
C9—N1—C1—O1	60.87 (18)	N1—C1—C8—C7	−29.16 (14)
C2—N1—C1—C12	155.41 (14)	O1—C1—C8—C7	82.58 (14)
C9—N1—C1—C12	−61.1 (2)	C12—C1—C8—C7	−152.16 (14)
C2—N1—C1—C8	31.23 (16)	N1—C1—C8—C11	−151.54 (13)
C9—N1—C1—C8	174.69 (14)	O1—C1—C8—C11	−39.80 (17)
C19—O1—C1—N1	−148.68 (13)	C12—C1—C8—C11	85.46 (17)
C19—O1—C1—C12	−25.67 (19)	N1—C1—C8—C10	86.38 (15)
C19—O1—C1—C8	101.54 (15)	O1—C1—C8—C10	−161.88 (12)
C1—N1—C2—C3	159.89 (16)	C12—C1—C8—C10	−36.62 (19)
C9—N1—C2—C3	16.9 (3)	N1—C1—C12—C13	139.09 (17)
C1—N1—C2—C7	−20.76 (18)	O1—C1—C12—C13	20.3 (2)
C9—N1—C2—C7	−163.71 (14)	C8—C1—C12—C13	−103.50 (19)
C7—C2—C3—C4	−1.8 (2)	C1—C12—C13—C14	−5.3 (3)
N1—C2—C3—C4	177.50 (16)	C12—C13—C14—C19	−6.3 (2)
C2—C3—C4—C5	0.5 (3)	C12—C13—C14—C15	175.75 (16)
C3—C4—C5—C6	0.5 (3)	C19—C14—C15—C16	0.5 (2)
C4—C5—C6—C7	−0.3 (2)	C13—C14—C15—C16	178.41 (15)
C5—C6—C7—C2	−1.0 (2)	C14—C15—C16—C17	−1.3 (2)
C5—C6—C7—C8	−177.85 (15)	C15—C16—C17—C18	0.2 (2)
C3—C2—C7—C6	2.1 (2)	C16—C17—C18—C19	1.8 (2)
N1—C2—C7—C6	−177.35 (14)	C1—O1—C19—C18	−166.22 (13)
C3—C2—C7—C8	179.55 (15)	C1—O1—C19—C14	16.5 (2)
N1—C2—C7—C8	0.14 (18)	C17—C18—C19—O1	180.00 (14)
C6—C7—C8—C11	−43.5 (2)	C17—C18—C19—C14	−2.7 (2)
C2—C7—C8—C11	139.34 (14)	C15—C14—C19—O1	178.75 (14)
C6—C7—C8—C10	79.1 (2)	C13—C14—C19—O1	0.7 (2)
C2—C7—C8—C10	−98.05 (15)	C15—C14—C19—C18	1.5 (2)
C6—C7—C8—C1	−164.75 (16)	C13—C14—C19—C18	−176.51 (14)

### (2) 1',3',3'-Trimethyl-4-[(E)-(1,3,3-trimethylindolin-2-ylidene)methyl]spiro[chroman-2,2'-indoline] Crystal data

**Table:**

C_31_H_34_N_2_O	*F*(000) = 968
*M**_r_* = 450.60	*D*_x_ = 1.239 Mg m^−^^3^
Monoclinic, *P*2_1_/*c*	Mo *K*α radiation, λ = 0.71073 Å
*a* = 14.1774 (11) Å	Cell parameters from 9967 reflections
*b* = 11.6019 (9) Å	θ = 2.3–31.0°
*c* = 16.2847 (17) Å	µ = 0.07 mm^−^^1^
β = 115.6129 (12)°	*T* = 90 K
*V* = 2415.4 (4) Å^3^	Plate, colorless
*Z* = 4	0.48 × 0.26 × 0.08 mm

### (2) 1',3',3'-Trimethyl-4-[(E)-(1,3,3-trimethylindolin-2-ylidene)methyl]spiro[chroman-2,2'-indoline] Data collection

**Table:**

Bruker DUO diffractometer	7680 independent reflections
Radiation source: fine focus sealed tube	6549 reflections with *I* > 2σ(*I*)
Detector resolution: 8.3 pixels mm^-1^	*R*_int_ = 0.026
ω scans	θ_max_ = 31.0°, θ_min_ = 2.4°
Absorption correction: multi-scan (SADABS; Bruker, 2014)	*h* = −20→20
*T*_min_ = 0.713, *T*_max_ = 0.746	*k* = −16→16
39237 measured reflections	*l* = −23→23

### (2) 1',3',3'-Trimethyl-4-[(E)-(1,3,3-trimethylindolin-2-ylidene)methyl]spiro[chroman-2,2'-indoline] Refinement

**Table:**

Refinement on *F*^2^	Primary atom site location: structure-invariant direct methods
Least-squares matrix: full	Secondary atom site location: difference Fourier map
*R*[*F*^2^ > 2σ(*F*^2^)] = 0.046	Hydrogen site location: inferred from neighbouring sites
*wR*(*F*^2^) = 0.124	H-atom parameters constrained
*S* = 1.03	*w* = 1/[σ^2^(*F*_o_^2^) + (0.0646*P*)^2^ + 0.9489*P*] where *P* = (*F*_o_^2^ + 2*F*_c_^2^)/3
7680 reflections	(Δ/σ)_max_ < 0.001
313 parameters	Δρ_max_ = 0.61 e Å^−^^3^
0 restraints	Δρ_min_ = −0.22 e Å^−^^3^

### (2) 1',3',3'-Trimethyl-4-[(E)-(1,3,3-trimethylindolin-2-ylidene)methyl]spiro[chroman-2,2'-indoline] Special details

**Table:**

Geometry. All esds (except the esd in the dihedral angle between two l.s. planes) are estimated using the full covariance matrix. The cell esds are taken into account individually in the estimation of esds in distances, angles and torsion angles; correlations between esds in cell parameters are only used when they are defined by crystal symmetry. An approximate (isotropic) treatment of cell esds is used for estimating esds involving l.s. planes.

### (2) 1',3',3'-Trimethyl-4-[(E)-(1,3,3-trimethylindolin-2-ylidene)methyl]spiro[chroman-2,2'-indoline] Fractional atomic coordinates and isotropic or equivalent isotropic displacement parameters (Å^2^)

**Table:**

	*x*	*y*	*z*	*U*_iso_*/*U*_eq_	
O1	0.34762 (5)	0.16535 (6)	0.32478 (5)	0.01634 (15)	
N1	0.40423 (7)	0.35186 (7)	0.37594 (6)	0.01547 (16)	
N2	−0.08953 (7)	0.35771 (8)	−0.01968 (6)	0.01639 (17)	
C1	0.31133 (7)	0.27916 (8)	0.33767 (7)	0.01376 (17)	
C2	0.45179 (8)	0.33748 (8)	0.47069 (7)	0.01494 (18)	
C3	0.55233 (8)	0.36930 (9)	0.53229 (7)	0.01845 (19)	
H3	0.5998	0.4030	0.5122	0.022*	
C4	0.58087 (9)	0.34990 (10)	0.62471 (7)	0.0212 (2)	
H4	0.6489	0.3712	0.6682	0.025*	
C5	0.51211 (9)	0.30028 (10)	0.65452 (8)	0.0231 (2)	
H5	0.5335	0.2875	0.7177	0.028*	
C6	0.41093 (9)	0.26899 (10)	0.59145 (7)	0.0211 (2)	
H6	0.3633	0.2355	0.6114	0.025*	
C7	0.38174 (8)	0.28778 (9)	0.49969 (7)	0.01572 (18)	
C8	0.27724 (7)	0.27236 (8)	0.41788 (7)	0.01457 (17)	
C9	0.47220 (8)	0.36495 (10)	0.33042 (7)	0.0195 (2)	
H9A	0.5114	0.4372	0.3498	0.029*	
H9B	0.4297	0.3664	0.2643	0.029*	
H9C	0.5212	0.3001	0.3464	0.029*	
C10	0.20944 (8)	0.37710 (10)	0.41676 (8)	0.0203 (2)	
H10A	0.2004	0.3784	0.4731	0.031*	
H10B	0.1408	0.3711	0.3644	0.031*	
H10C	0.2440	0.4482	0.4120	0.031*	
C11	0.22236 (8)	0.16035 (10)	0.42081 (7)	0.0203 (2)	
H11A	0.2120	0.1582	0.4765	0.030*	
H11B	0.2655	0.0946	0.4202	0.030*	
H11C	0.1544	0.1563	0.3676	0.030*	
C12	0.23105 (8)	0.32528 (9)	0.24650 (7)	0.01534 (18)	
H12A	0.2665	0.3408	0.2070	0.018*	
H12B	0.2034	0.3994	0.2569	0.018*	
C13	0.13889 (7)	0.24303 (8)	0.19642 (7)	0.01457 (17)	
H13	0.0967	0.2367	0.2320	0.017*	
C14	0.18259 (7)	0.12496 (8)	0.19179 (6)	0.01406 (17)	
C15	0.12593 (8)	0.04410 (9)	0.12446 (7)	0.01773 (19)	
H15	0.0575	0.0634	0.0805	0.021*	
C16	0.16694 (8)	−0.06372 (9)	0.12009 (7)	0.0192 (2)	
H16	0.1272	−0.1169	0.0736	0.023*	
C17	0.26725 (8)	−0.09253 (9)	0.18494 (8)	0.0198 (2)	
H17	0.2960	−0.1658	0.1827	0.024*	
C18	0.32501 (8)	−0.01440 (9)	0.25269 (7)	0.01735 (19)	
H18	0.3930	−0.0344	0.2969	0.021*	
C19	0.28304 (7)	0.09388 (8)	0.25583 (7)	0.01425 (17)	
C20	0.06994 (8)	0.28955 (9)	0.10287 (7)	0.01557 (18)	
H20	0.1026	0.3022	0.0635	0.019*	
C21	−0.03249 (7)	0.31510 (8)	0.06905 (6)	0.01364 (17)	
C22	−0.19342 (8)	0.37872 (8)	−0.03783 (7)	0.01475 (18)	
C23	−0.27463 (8)	0.42123 (9)	−0.11683 (7)	0.01866 (19)	
H23	−0.2638	0.4406	−0.1688	0.022*	
C24	−0.37283 (8)	0.43437 (10)	−0.11672 (8)	0.0213 (2)	
H24	−0.4295	0.4630	−0.1698	0.026*	
C25	−0.38953 (8)	0.40669 (10)	−0.04102 (8)	0.0211 (2)	
H25	−0.4569	0.4165	−0.0427	0.025*	
C26	−0.30669 (8)	0.36425 (9)	0.03782 (7)	0.01789 (19)	
H26	−0.3173	0.3453	0.0899	0.021*	
C27	−0.20940 (7)	0.35039 (8)	0.03879 (7)	0.01449 (18)	
C28	−0.10793 (7)	0.30639 (8)	0.11424 (6)	0.01381 (17)	
C29	−0.07525 (8)	0.38417 (10)	0.19859 (7)	0.0196 (2)	
H29A	−0.0588	0.4614	0.1842	0.029*	
H29B	−0.0134	0.3515	0.2488	0.029*	
H29C	−0.1327	0.3891	0.2166	0.029*	
C30	−0.12197 (9)	0.18061 (9)	0.13746 (8)	0.0211 (2)	
H30A	−0.1753	0.1774	0.1606	0.032*	
H30B	−0.0555	0.1515	0.1840	0.032*	
H30C	−0.1441	0.1331	0.0825	0.032*	
C31	−0.04513 (9)	0.37235 (10)	−0.08373 (7)	0.0209 (2)	
H31A	−0.0985	0.4030	−0.1411	0.031*	
H31B	−0.0205	0.2977	−0.0950	0.031*	
H31C	0.0138	0.4263	−0.0586	0.031*	

### (2) 1',3',3'-Trimethyl-4-[(E)-(1,3,3-trimethylindolin-2-ylidene)methyl]spiro[chroman-2,2'-indoline] Atomic displacement parameters (Å^2^)

**Table:**

	*U*^11^	*U*^22^	*U*^33^	*U*^12^	*U*^13^	*U*^23^
O1	0.0140 (3)	0.0146 (3)	0.0178 (3)	0.0019 (2)	0.0043 (3)	−0.0028 (3)
N1	0.0150 (4)	0.0173 (4)	0.0146 (4)	−0.0025 (3)	0.0069 (3)	−0.0006 (3)
N2	0.0156 (4)	0.0212 (4)	0.0129 (4)	0.0024 (3)	0.0068 (3)	0.0041 (3)
C1	0.0142 (4)	0.0130 (4)	0.0143 (4)	0.0015 (3)	0.0064 (3)	0.0002 (3)
C2	0.0160 (4)	0.0139 (4)	0.0147 (4)	0.0016 (3)	0.0065 (3)	−0.0006 (3)
C3	0.0164 (4)	0.0180 (4)	0.0202 (5)	−0.0006 (3)	0.0072 (4)	−0.0026 (4)
C4	0.0187 (5)	0.0217 (5)	0.0187 (5)	0.0013 (4)	0.0041 (4)	−0.0034 (4)
C5	0.0246 (5)	0.0263 (5)	0.0149 (4)	0.0016 (4)	0.0054 (4)	0.0008 (4)
C6	0.0226 (5)	0.0247 (5)	0.0168 (5)	0.0005 (4)	0.0091 (4)	0.0026 (4)
C7	0.0161 (4)	0.0156 (4)	0.0154 (4)	0.0023 (3)	0.0067 (3)	0.0013 (3)
C8	0.0149 (4)	0.0159 (4)	0.0141 (4)	0.0011 (3)	0.0074 (3)	0.0014 (3)
C9	0.0186 (4)	0.0232 (5)	0.0200 (5)	−0.0021 (4)	0.0115 (4)	0.0004 (4)
C10	0.0187 (4)	0.0227 (5)	0.0201 (5)	0.0041 (4)	0.0088 (4)	−0.0019 (4)
C11	0.0212 (5)	0.0220 (5)	0.0191 (5)	−0.0041 (4)	0.0100 (4)	0.0017 (4)
C12	0.0160 (4)	0.0150 (4)	0.0140 (4)	0.0010 (3)	0.0055 (3)	0.0010 (3)
C13	0.0142 (4)	0.0156 (4)	0.0139 (4)	0.0021 (3)	0.0061 (3)	0.0014 (3)
C14	0.0140 (4)	0.0154 (4)	0.0135 (4)	0.0009 (3)	0.0066 (3)	0.0005 (3)
C15	0.0174 (4)	0.0191 (5)	0.0153 (4)	−0.0009 (3)	0.0057 (3)	0.0000 (3)
C16	0.0231 (5)	0.0174 (4)	0.0172 (4)	−0.0030 (4)	0.0088 (4)	−0.0038 (4)
C17	0.0218 (5)	0.0169 (4)	0.0230 (5)	0.0008 (4)	0.0118 (4)	−0.0025 (4)
C18	0.0155 (4)	0.0172 (4)	0.0204 (5)	0.0021 (3)	0.0087 (4)	−0.0004 (4)
C19	0.0136 (4)	0.0150 (4)	0.0153 (4)	0.0001 (3)	0.0074 (3)	−0.0006 (3)
C20	0.0157 (4)	0.0181 (4)	0.0136 (4)	0.0018 (3)	0.0069 (3)	0.0017 (3)
C21	0.0164 (4)	0.0132 (4)	0.0117 (4)	0.0002 (3)	0.0064 (3)	0.0005 (3)
C22	0.0154 (4)	0.0132 (4)	0.0141 (4)	−0.0007 (3)	0.0050 (3)	−0.0006 (3)
C23	0.0199 (5)	0.0180 (5)	0.0144 (4)	0.0000 (4)	0.0039 (4)	0.0014 (3)
C24	0.0173 (4)	0.0200 (5)	0.0200 (5)	0.0008 (4)	0.0018 (4)	0.0007 (4)
C25	0.0140 (4)	0.0212 (5)	0.0241 (5)	−0.0007 (4)	0.0046 (4)	−0.0007 (4)
C26	0.0152 (4)	0.0188 (5)	0.0190 (5)	−0.0024 (3)	0.0067 (4)	−0.0009 (4)
C27	0.0141 (4)	0.0133 (4)	0.0148 (4)	−0.0013 (3)	0.0051 (3)	−0.0004 (3)
C28	0.0144 (4)	0.0148 (4)	0.0130 (4)	0.0005 (3)	0.0066 (3)	0.0013 (3)
C29	0.0169 (4)	0.0265 (5)	0.0143 (4)	0.0026 (4)	0.0057 (4)	−0.0025 (4)
C30	0.0210 (5)	0.0184 (5)	0.0255 (5)	0.0000 (4)	0.0116 (4)	0.0067 (4)
C31	0.0222 (5)	0.0276 (5)	0.0158 (4)	0.0015 (4)	0.0111 (4)	0.0042 (4)

### (2) 1',3',3'-Trimethyl-4-[(E)-(1,3,3-trimethylindolin-2-ylidene)methyl]spiro[chroman-2,2'-indoline] Geometric parameters (Å, º)

**Table:**

O1—C19	1.3776 (12)	C13—H13	1.0000
O1—C1	1.4648 (12)	C14—C19	1.4005 (13)
N1—C2	1.4013 (13)	C14—C15	1.4022 (14)
N1—C9	1.4556 (13)	C15—C16	1.3942 (15)
N1—C1	1.4577 (13)	C15—H15	0.9500
N2—C22	1.3928 (13)	C16—C17	1.3967 (15)
N2—C21	1.4056 (12)	C16—H16	0.9500
N2—C31	1.4425 (13)	C17—C18	1.3887 (15)
C1—C12	1.5247 (13)	C17—H17	0.9500
C1—C8	1.5785 (14)	C18—C19	1.4004 (14)
C2—C3	1.3921 (14)	C18—H18	0.9500
C2—C7	1.3956 (14)	C20—C21	1.3448 (13)
C3—C4	1.3975 (15)	C20—H20	0.9500
C3—H3	0.9500	C21—C28	1.5415 (13)
C4—C5	1.3881 (17)	C22—C23	1.3941 (13)
C4—H4	0.9500	C22—C27	1.4002 (14)
C5—C6	1.4037 (16)	C23—C24	1.4013 (15)
C5—H5	0.9500	C23—H23	0.9500
C6—C7	1.3845 (14)	C24—C25	1.3896 (16)
C6—H6	0.9500	C24—H24	0.9500
C7—C8	1.5150 (14)	C25—C26	1.4023 (14)
C8—C11	1.5260 (14)	C25—H25	0.9500
C8—C10	1.5446 (14)	C26—C27	1.3820 (14)
C9—H9A	0.9800	C26—H26	0.9500
C9—H9B	0.9800	C27—C28	1.5204 (13)
C9—H9C	0.9800	C28—C29	1.5381 (14)
C10—H10A	0.9800	C28—C30	1.5418 (14)
C10—H10B	0.9800	C29—H29A	0.9800
C10—H10C	0.9800	C29—H29B	0.9800
C11—H11A	0.9800	C29—H29C	0.9800
C11—H11B	0.9800	C30—H30A	0.9800
C11—H11C	0.9800	C30—H30B	0.9800
C12—C13	1.5367 (14)	C30—H30C	0.9800
C12—H12A	0.9900	C31—H31A	0.9800
C12—H12B	0.9900	C31—H31B	0.9800
C13—C20	1.5100 (13)	C31—H31C	0.9800
C13—C14	1.5186 (14)		
			
C19—O1—C1	120.56 (7)	C19—C14—C15	117.67 (9)
C2—N1—C9	117.63 (8)	C19—C14—C13	120.04 (8)
C2—N1—C1	108.51 (8)	C15—C14—C13	122.29 (9)
C9—N1—C1	121.16 (8)	C16—C15—C14	121.98 (9)
C22—N2—C21	111.43 (8)	C16—C15—H15	119.0
C22—N2—C31	125.31 (8)	C14—C15—H15	119.0
C21—N2—C31	123.21 (8)	C15—C16—C17	119.14 (9)
N1—C1—O1	105.93 (7)	C15—C16—H16	120.4
N1—C1—C12	111.68 (8)	C17—C16—H16	120.4
O1—C1—C12	109.72 (8)	C18—C17—C16	120.16 (10)
N1—C1—C8	102.62 (8)	C18—C17—H17	119.9
O1—C1—C8	109.01 (7)	C16—C17—H17	119.9
C12—C1—C8	117.17 (8)	C17—C18—C19	120.04 (9)
C3—C2—C7	121.49 (9)	C17—C18—H18	120.0
C3—C2—N1	128.12 (9)	C19—C18—H18	120.0
C7—C2—N1	110.35 (8)	O1—C19—C18	115.25 (8)
C2—C3—C4	117.58 (10)	O1—C19—C14	123.75 (9)
C2—C3—H3	121.2	C18—C19—C14	121.00 (9)
C4—C3—H3	121.2	C21—C20—C13	127.27 (9)
C5—C4—C3	121.55 (10)	C21—C20—H20	116.4
C5—C4—H4	119.2	C13—C20—H20	116.4
C3—C4—H4	119.2	C20—C21—N2	122.55 (9)
C4—C5—C6	120.10 (10)	C20—C21—C28	129.73 (9)
C4—C5—H5	120.0	N2—C21—C28	107.71 (8)
C6—C5—H5	120.0	N2—C22—C23	129.31 (9)
C7—C6—C5	118.89 (10)	N2—C22—C27	109.48 (8)
C7—C6—H6	120.6	C23—C22—C27	121.21 (9)
C5—C6—H6	120.6	C22—C23—C24	117.51 (10)
C6—C7—C2	120.39 (9)	C22—C23—H23	121.2
C6—C7—C8	130.77 (9)	C24—C23—H23	121.2
C2—C7—C8	108.64 (8)	C25—C24—C23	121.77 (10)
C7—C8—C11	113.02 (8)	C25—C24—H24	119.1
C7—C8—C10	106.52 (8)	C23—C24—H24	119.1
C11—C8—C10	110.32 (8)	C24—C25—C26	119.79 (10)
C7—C8—C1	100.90 (8)	C24—C25—H25	120.1
C11—C8—C1	114.27 (8)	C26—C25—H25	120.1
C10—C8—C1	111.26 (8)	C27—C26—C25	119.21 (10)
N1—C9—H9A	109.5	C27—C26—H26	120.4
N1—C9—H9B	109.5	C25—C26—H26	120.4
H9A—C9—H9B	109.5	C26—C27—C22	120.51 (9)
N1—C9—H9C	109.5	C26—C27—C28	129.74 (9)
H9A—C9—H9C	109.5	C22—C27—C28	109.75 (8)
H9B—C9—H9C	109.5	C27—C28—C29	109.83 (8)
C8—C10—H10A	109.5	C27—C28—C21	101.63 (8)
C8—C10—H10B	109.5	C29—C28—C21	112.62 (8)
H10A—C10—H10B	109.5	C27—C28—C30	109.79 (8)
C8—C10—H10C	109.5	C29—C28—C30	110.93 (8)
H10A—C10—H10C	109.5	C21—C28—C30	111.65 (8)
H10B—C10—H10C	109.5	C28—C29—H29A	109.5
C8—C11—H11A	109.5	C28—C29—H29B	109.5
C8—C11—H11B	109.5	H29A—C29—H29B	109.5
H11A—C11—H11B	109.5	C28—C29—H29C	109.5
C8—C11—H11C	109.5	H29A—C29—H29C	109.5
H11A—C11—H11C	109.5	H29B—C29—H29C	109.5
H11B—C11—H11C	109.5	C28—C30—H30A	109.5
C1—C12—C13	113.85 (8)	C28—C30—H30B	109.5
C1—C12—H12A	108.8	H30A—C30—H30B	109.5
C13—C12—H12A	108.8	C28—C30—H30C	109.5
C1—C12—H12B	108.8	H30A—C30—H30C	109.5
C13—C12—H12B	108.8	H30B—C30—H30C	109.5
H12A—C12—H12B	107.7	N2—C31—H31A	109.5
C20—C13—C14	111.87 (8)	N2—C31—H31B	109.5
C20—C13—C12	110.16 (8)	H31A—C31—H31B	109.5
C14—C13—C12	108.36 (8)	N2—C31—H31C	109.5
C20—C13—H13	108.8	H31A—C31—H31C	109.5
C14—C13—H13	108.8	H31B—C31—H31C	109.5
C12—C13—H13	108.8		
			
C2—N1—C1—O1	−85.70 (9)	C19—C14—C15—C16	−0.24 (15)
C9—N1—C1—O1	55.02 (11)	C13—C14—C15—C16	179.36 (9)
C2—N1—C1—C12	154.89 (8)	C14—C15—C16—C17	0.36 (16)
C9—N1—C1—C12	−64.39 (11)	C15—C16—C17—C18	−0.04 (16)
C2—N1—C1—C8	28.56 (10)	C16—C17—C18—C19	−0.40 (16)
C9—N1—C1—C8	169.28 (8)	C1—O1—C19—C18	−177.53 (8)
C19—O1—C1—N1	−149.92 (8)	C1—O1—C19—C14	3.06 (14)
C19—O1—C1—C12	−29.24 (11)	C17—C18—C19—O1	−178.90 (9)
C19—O1—C1—C8	100.29 (10)	C17—C18—C19—C14	0.53 (15)
C9—N1—C2—C3	22.49 (15)	C15—C14—C19—O1	179.16 (9)
C1—N1—C2—C3	164.79 (10)	C13—C14—C19—O1	−0.44 (14)
C9—N1—C2—C7	−159.66 (9)	C15—C14—C19—C18	−0.21 (14)
C1—N1—C2—C7	−17.36 (11)	C13—C14—C19—C18	−179.82 (9)
C7—C2—C3—C4	−0.12 (15)	C14—C13—C20—C21	117.80 (11)
N1—C2—C3—C4	177.51 (10)	C12—C13—C20—C21	−121.61 (11)
C2—C3—C4—C5	0.29 (16)	C13—C20—C21—N2	−179.70 (9)
C3—C4—C5—C6	−0.46 (17)	C13—C20—C21—C28	0.79 (18)
C4—C5—C6—C7	0.44 (17)	C22—N2—C21—C20	−179.21 (9)
C5—C6—C7—C2	−0.28 (16)	C31—N2—C21—C20	3.22 (16)
C5—C6—C7—C8	−174.41 (10)	C22—N2—C21—C28	0.39 (11)
C3—C2—C7—C6	0.12 (15)	C31—N2—C21—C28	−177.17 (9)
N1—C2—C7—C6	−177.89 (9)	C21—N2—C22—C23	179.71 (10)
C3—C2—C7—C8	175.44 (9)	C31—N2—C22—C23	−2.79 (17)
N1—C2—C7—C8	−2.58 (11)	C21—N2—C22—C27	−0.10 (12)
C6—C7—C8—C11	−43.62 (15)	C31—N2—C22—C27	177.40 (10)
C2—C7—C8—C11	141.72 (9)	N2—C22—C23—C24	−179.88 (10)
C6—C7—C8—C10	77.68 (13)	C27—C22—C23—C24	−0.09 (15)
C2—C7—C8—C10	−96.98 (10)	C22—C23—C24—C25	0.19 (16)
C6—C7—C8—C1	−166.08 (11)	C23—C24—C25—C26	−0.08 (17)
C2—C7—C8—C1	19.26 (10)	C24—C25—C26—C27	−0.14 (16)
N1—C1—C8—C7	−28.15 (9)	C25—C26—C27—C22	0.23 (15)
O1—C1—C8—C7	83.84 (9)	C25—C26—C27—C28	−179.83 (10)
C12—C1—C8—C7	−150.86 (8)	N2—C22—C27—C26	179.71 (9)
N1—C1—C8—C11	−149.73 (8)	C23—C22—C27—C26	−0.12 (15)
O1—C1—C8—C11	−37.73 (11)	N2—C22—C27—C28	−0.24 (11)
C12—C1—C8—C11	87.57 (10)	C23—C22—C27—C28	179.93 (9)
N1—C1—C8—C10	84.53 (9)	C26—C27—C28—C29	−60.05 (13)
O1—C1—C8—C10	−163.48 (8)	C22—C27—C28—C29	119.89 (9)
C12—C1—C8—C10	−38.18 (11)	C26—C27—C28—C21	−179.50 (10)
N1—C1—C12—C13	171.51 (8)	C22—C27—C28—C21	0.44 (10)
O1—C1—C12—C13	54.37 (10)	C26—C27—C28—C30	62.18 (13)
C8—C1—C12—C13	−70.57 (11)	C22—C27—C28—C30	−117.88 (9)
C1—C12—C13—C20	−173.83 (8)	C20—C21—C28—C27	179.07 (10)
C1—C12—C13—C14	−51.16 (11)	N2—C21—C28—C27	−0.49 (10)
C20—C13—C14—C19	145.65 (9)	C20—C21—C28—C29	61.62 (14)
C12—C13—C14—C19	24.02 (12)	N2—C21—C28—C29	−117.94 (9)
C20—C13—C14—C15	−33.94 (13)	C20—C21—C28—C30	−63.95 (14)
C12—C13—C14—C15	−155.57 (9)	N2—C21—C28—C30	116.48 (9)
